# Removal behavior and chemical speciation distributions of heavy metals in sewage sludge during bioleaching and combined bioleaching/Fenton-like processes

**DOI:** 10.1038/s41598-021-94216-2

**Published:** 2021-07-21

**Authors:** Chunsheng Qiu, Shangyu Xie, Nannan Liu, Kequan Meng, Chenchen Wang, Dong Wang, Shaopo Wang

**Affiliations:** 1 School of Environmental and Municipal Engineering, Tianjin Chengjian University, Tianjin, 300384 People’s Republic of China; 2 Tianjin Key Laboratory of Aqueous Science and Technology, Tianjin Chengjian University, No. 26, Jinjing Road, Xiqing District, Tianjin, 300384 People’s Republic of China; 3 CNOOC Ener Tech-Drilling & Production Co., Tianjin, 300452 People’s Republic of China

**Keywords:** Ecology, Environmental sciences

## Abstract

The removal and chemical speciation changes of heavy metals in the sewage sludge during the single bioleaching and combined bioleaching/Fenton-like processes were compared in this study. The improvement in the dewaterability of the treated sludge was also investigated. The single bioleaching led to a removal of Zn, Cu, Cd, Cr, Mn, Ni, As and Pb of 67.28%, 50.78%, 64.86%, 6.32%, 56.15%, 49.83%, 20.78% and 10.52% in 10 days, respectively. The chemical speciation analysis showed that the solubilization of heavy metals in mobile forms (exchangeable/acid soluble and reducible forms) and oxidizable form was the main reason for their removal. Subsequent Fenton-like treatment was carried out at different bioleaching stages when the bioleached sludge dropped to certain pH values (4.5, 4.0 and 3.0), by adding H_2_O_2_ at different dosages. The highest removal ratio of Zn, Cu, Cd, Cr, Mn and Ni could reach 75.53%, 52.17%, 71.91%, 11.63%, 66.29% and 65.19% after combined bioleaching/Fenton-like process, respectively, with appropriate pH and H_2_O_2_ dosages in less than 6 days. The solubilization efficiencies of these heavy metals in mobile forms were further improved by Fenton-like treatment. The removal efficiencies of As and Pb decreased due to their transformation into insoluble forms (mostly residual fraction) after Fenton treatment. The capillary suction times (CST) of the raw sludge (98.7 s) decreased by 79.43% after bioleaching and 87.44% after combined process, respectively.

## Introduction

The amount of sewage sludge is increasing fast worldwide due to the growing wastewater quantity and stringent environmental regulations. Land application of sewage sludge after appropriate treatment has become an important disposal alternative due to its high content of organic and inorganic nutrients^1–3^. However, the presence of contaminants in sewage sludge, especially heavy metals which are toxic and non-biodegradable, limits its utilization as fertilizer or soil amendment^4–7^.

Bioleaching has been considered as an environmentally friendly and promising method for the removal of heavy metals from contaminated sediment and sludge^5,8,9^. Chemoautotrophic iron-oxidizing and sulfur-oxidizing strains can generate sulfuric acid or ferric ions using S^0^ or Fe^2+^ as substrate, leading to a highly acidic environment and generation of soluble metal sulfate, and thus enable the metals to move from solid phase to liquid phase^5,10^. The rate of microbial sulfur and ferrous ion oxidation is the limiting step governing the efficiency of bioleaching process, and it has been reported that long time length (6–12 days) is usually required to obtain high solubilization efficiency and thus makes this process non-economically viable^11,12^.

Fenton and Fenton-like reaction as advanced oxidation processes have been widely applied to conditioning sludge^13,14^. Powerful oxidizing agent hydroxyl radical can be produced under acidic condition through the reaction of hydrogen peroxide (H_2_O_2_) catalyzed by ferrous (Fe^2+^) or ferric (Fe^3+^) ions. Previous studies indicated that Fenton oxidation could improve sludge dewatering by degrading extracellular polymeric substances (EPS) and releasing bound water, and enhance anaerobic biodegradability of the sewage sludge^13–15^. Fenton oxidation was also reported to efficiently release heavy metals from sewage sludge^16^. However, due to the high buffering capacity of the sewage sludge, large amounts of inorganic acid are required to achieve the desired acidic condition for Fenton reaction, leading to high operational cost. During the bioleaching process, the pH value of the bioleached sludge could decline to an optimal value for Fenton oxidation, and ferrous ion added as substrate for iron-oxidizing bacteria^17^ could also act as catalyst for Fenton reaction. The subsequent Fenton reaction after bioleaching can accelerate the heavy metals dissolution and sludge dewatering, and thus shorten the operating period for leaching. However, studies on the combination of bioleaching and Fenton reaction are yet limited. Fontmorin and Sillanpaaand investigated the efficiency of combined bioleaching/Fenton-like process for heavy metals removal and dewaterability improvement of the sludge^10^. Zhu et al. obtained high removal efficiency of Cu, Zn, Pb and Cd after 5-day bioleaching and subsequent Fenton like treatment^18^. However, little attention was paid to the change of chemical speciation of heavy metals. According to BCR extraction procedure^19^, the chemical speciation of metals could be classified as exchangeable/acid soluble, reducible, oxidizable and residual forms. The negative impact of heavy metals in sludge is largely determined by their chemical speciation and distributions^20–22^. However, the chemical form distributions of heavy metals in sewage sludge during the combined process of bioleaching and Fenton oxidation are still poorly understood. In addition, the efficiency of Fenton oxidation is mainly depended on the reaction condition, therefore, it is also crucial to investigate the influence of reaction pH and H_2_O_2_ dosage in the combined bioleaching/Fenton-like process, which has also been neglected in previous studies.

In this study, the single bioleaching and combined bioleaching/Fenton-like processes were applied to remove heavy metals (Zn, Cu, Cd, Cr, Mn, Ni, As and Pb) from sewage sludge. The solubilization efficiency, chemical speciation changes of heavy metals, and dewaterability of sludge during these two processes were compared. The effect of H_2_O_2_ dose and reaction pH during Fenton-like process was also analyzed.

## Materials and methods

### Sludge samples

The sludge samples were collected from a local municipal sludge treatment plant in Tianjin, China, and then stored at 4 °C for further use. Prior to the bioleaching experiments, total solid (TS) of the raw sludge was adjusted to 4.0% with deionized water. Total concentrations of the heavy metals in the raw sludge and the control standards of pollutants in sludge for agricultural use of China (National Standard GB 4284-2018) are listed in Table 1.

**Table 1:** Total concentrations of heavy metals in the raw sludge.

Element (mg/kg-dry solids)	Concentration	Control standards (GB 2484–2018, A grade)
Zn	2661.90 ± 117.08	≤ 1200
Cu	788.66 ± 25.22	≤ 500
Cd	6.11 ± 0.31	≤ 3
Cr	437.98 ± 22.25	≤ 500
Mn	518.79 ± 27.58	–
Ni	92.78 ± 2.36	≤ 100
As	33.77 ± 1.05	≤ 30
Pb	61.85 ± 2.52	≤ 300

### Enrichment of indigenous iron-oxidizing bacteria

The mixed culture of iron-oxidizing bacteria was used as inoculum for the bioleaching experiments, and the enrichment culture was carried out following the process described in detail in our previous study^23^. Fresh sludge sample from the thickening tank of a local municipal wastewater treatment plant was used as the seed sludge for enrichment culture of indigenous iron-oxidizing bacteria^24^.

### Bioleaching experiments

The procedures of bioleaching experiments were proceeded according to the process described in our previous study^23^. 300 mL of sludge sample was mixed with 5% (v/v) inocula and 4.00 g/L Fe^2+^ (FeSO_4_) as iron substrate. The conical flasks were placed in water bath shakers set at 150 rpm and 28 °C. The pH and oxidation–reduction potential (ORP) of the leaching sludge were monitored over time. 20 mL of the sludge samples was taken out every 48 h for heavy metals analysis.

### Combined bioleaching/Fenton-like experiments

The combination of bioleaching and Fenton-like reaction consisted of two steps. The bioleaching step was carried out following the procedures described in “Bioleaching experiments” section. When the pH of the leaching sludge dropped to 4.5, 4.0 and 3.0, the Fenton-like reaction step was performed by adding H_2_O_2_ into the bioleached sludge with dosage of 5.0, 8.0, 11.0, 13.0 and 15.0 g/L.

All experiments were carried out in triplicate.

### Analysis

The pH, ORP and TS contents of the sludge were determined according to the standard methods^25^. Capillary suction time (CST) was measured by CST analyzer (304M, Triton).

The total concentrations of heavy metals were measured using inductively coupled plasma mass spectrometry (ICP-MS, Agilent 7700), and the sample pretreatment procedure was proceeded according to our previous study^23^. The chemical forms of the heavy metals investigated were analyzed using the improved BCR procedures described by Rauret et al.^19^.

## Results and discussion

### Bioleaching process

#### Variation of pH and ORP during bioleaching process

**Figure 1:**
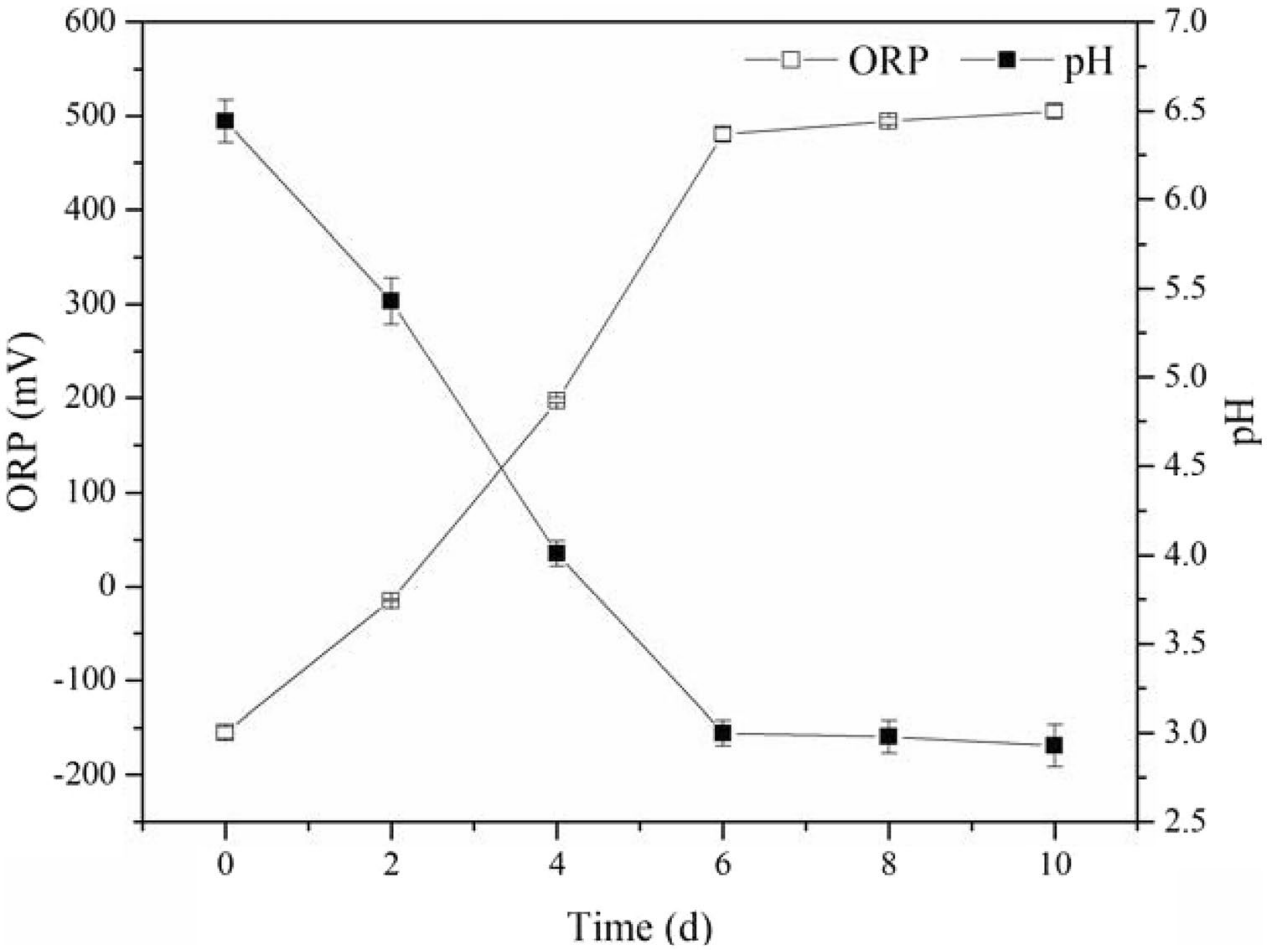
Variation of pH and ORP during bioleaching process.

pH and ORP of the sludge are widely known to be the important parameters influencing heavy metal solubilization during bioleaching process, as well as the activity of iron-oxidizing microorganisms^10,26,27^. The variation of sludge pH and ORP during the single bioleaching process is presented in Fig. 1.

An appropriate pH could enhance the activities of microbes, affecting the release of metals and the stability of metal ions in the liquid phase^5^. As shown in Fig. 1, the pH value of sewage sludge quickly decreased from 6.44 to 3.07 in the first 6 days, due to the oxidation of Fe^2+^ and metal sulfides, the production of sulfuric acid, ferric hydroxide and jarosite from the hydrolysis of Fe^3+^^18^. Then the pH gradually decreased to 2.89 on the 10th day. The change of ORP followed an opposite trend. ORP value of the sludge rapidly increased from − 155.6 mV to 480.0 mV in the first 6 days, then to 505.0 mV in the following 4 days, due to the oxidation of Fe^2+^ to Fe^3+^ by leaching microorganisms.

#### Heavy metals solubilization and chemical speciation distribution during bioleaching process

**Figure 2:**
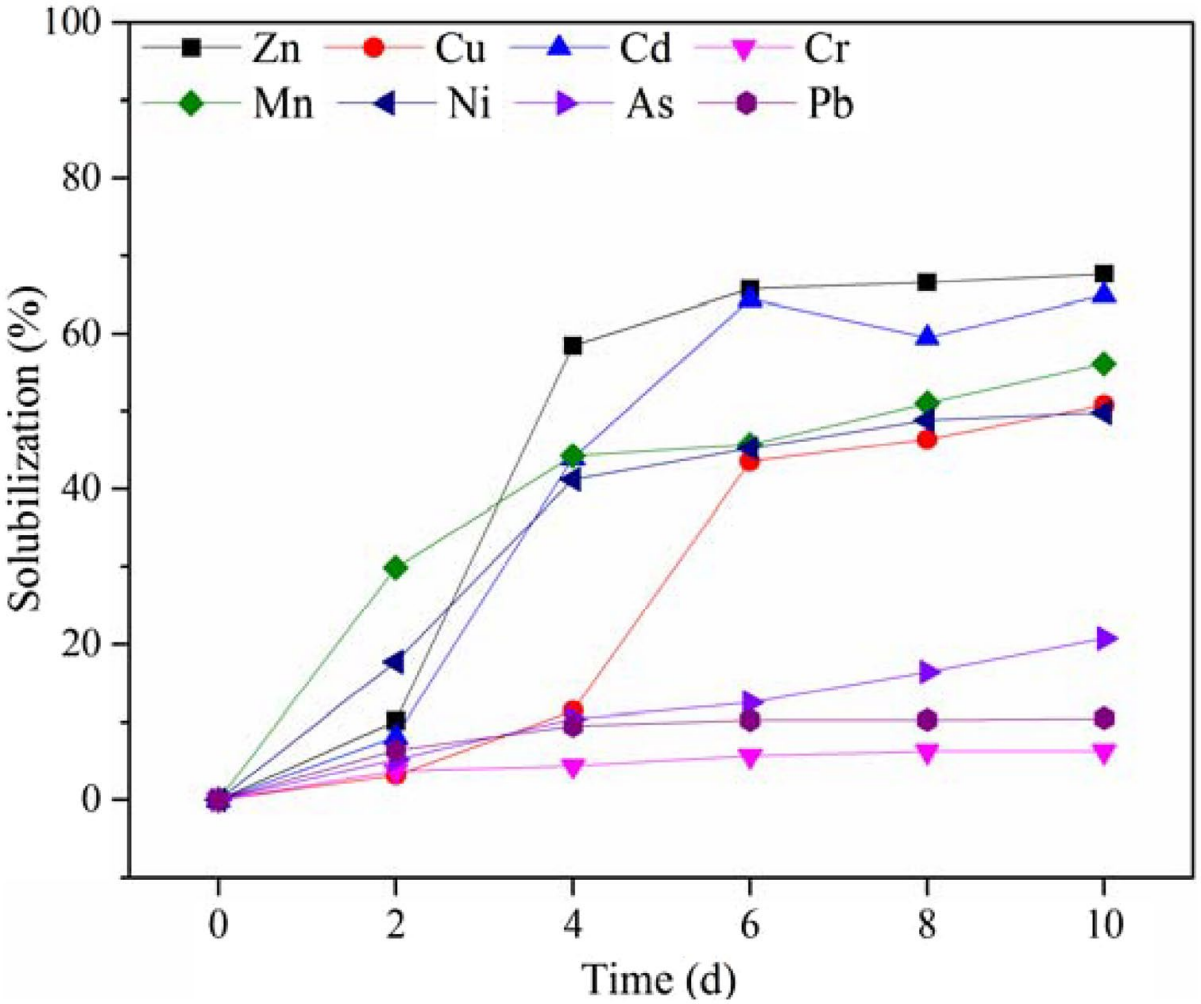
Removal of heavy metals during bioleaching process.

**Figure 3:**
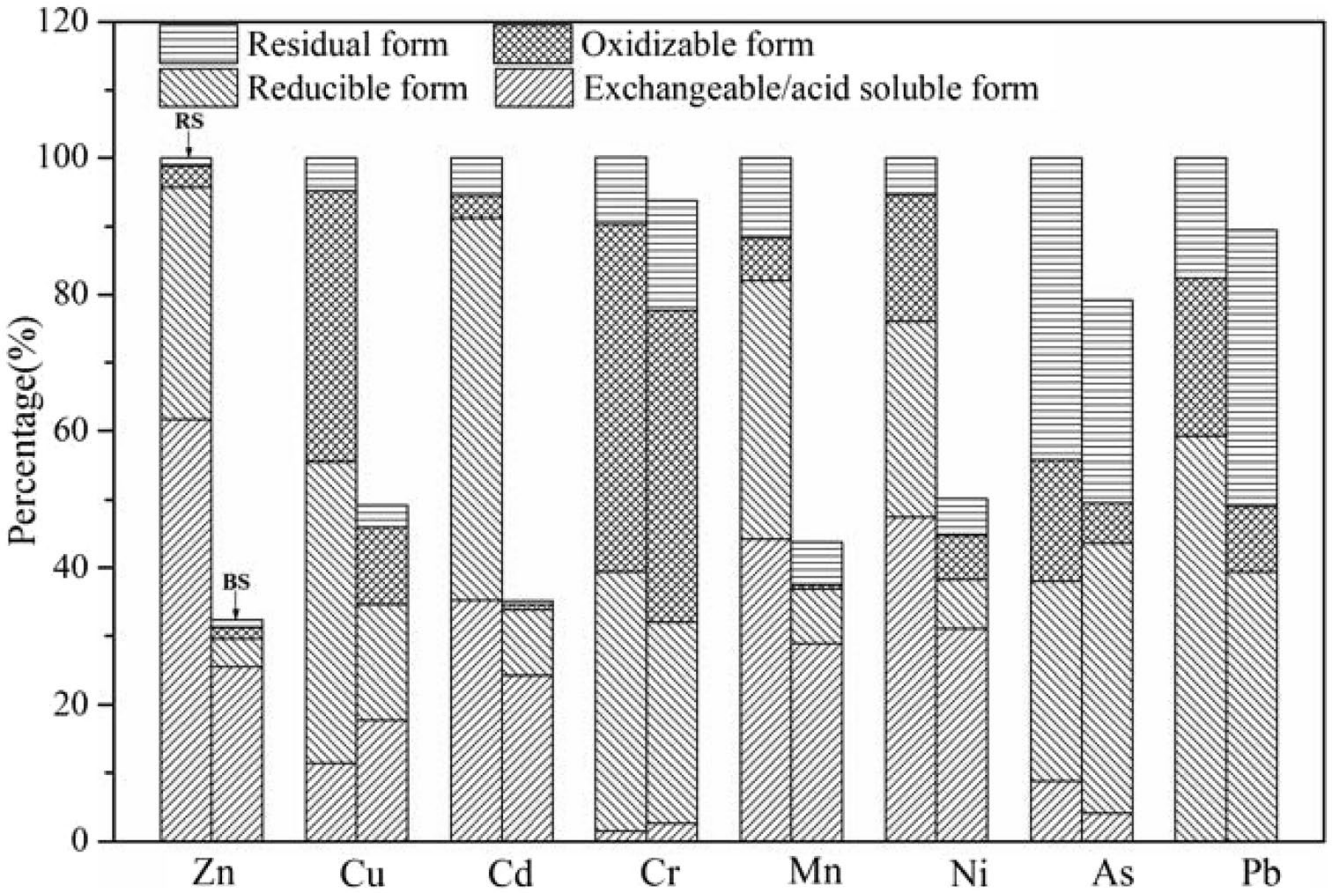
Chemical speciation distributions of heavy metals in raw sludge and bioleached sludge, total concentration of heavy metals in the raw sludge was set as 100% (*RS* raw sludge, *BS* bioleached sludge).

The removal of heavy metals during bioleaching process and the distribution of chemical fractions of heavy metals before and after bioleaching are presented in Figs. 2 and 3, respectively. The single bioleaching led to the removal of Zn, Cu, Cd, Cr, Mn, Ni, As and Pb of 67.28%, 50.78%, 64.86%, 6.32%, 56.15%, 49.83%, 20.78% and 10.52% in 10 days, respectively. The solubilization efficiency was highly related to the evolution of pH and ORP, the chemical fraction distributions and the nature of heavy metals.

Figure 2 illustrated that Zn had the highest solubilization and removal efficiency. It was found that below the threshold pH of 6–6.5, Zn could be dissolved^28^. Thus, the dissolving out of Zn had started at the beginning of leaching experiment with a removal percentage of 10.15% on the 2nd day. Yet the quick solubilization of Zn was observed from the 4th day (pH 4.01). And until the 6th day (pH 3.00) when the solubilization percentage of Zn was 65.71%, the leaching rate of Zn was slowed down due to the stable pH. In the raw sludge, Zn mainly existed in mobile forms (exchangeable/acid soluble and reducible forms) as shown in Fig. 3. After bioleaching, the solubilization efficiencies of Zn in exchangeable/acid soluble form and reducible form was 58.66% and 87.93%, respectively. Meanwhile, 48.27% of Zn in oxidizable form was also dissolved out due to the oxidation of metal sulfide and loss of sludge organic matter. However, Zn in residual form remained almost unchanged in the bioleached sludge due to its high stability.

It has been pointed out that Cu could be rapidly solubilized below pH of 3.7 or under a high ORP condition^29^. As shown in Fig. 2, in the first 4 days, the solubilization efficiency of Cu was relatively low (11.44%). The removal rate of Cu increased rapidly to 43.54% on the 6th day due to the increase of ORP (480 mV). The proportion of Cu in exchangeable/acid soluble form increased by 55.16% after bioleaching, probably because the solubilized Cu^2+^ was re-adsorbed on the EPS of sludge cells^30,31^. Most of Cu was present in reducible and oxidizable forms in the raw sludge as shown in Fig. 3, because the complexation of copper and organic materials was relatively stable^30,32,33^. The removal percentages of Cu in reducible and oxidizable forms were 71.11% and 61.83% after bioleaching, respectively, which was the main reason for Cu removal.

Cd could be solubilized rapidly under acidic conditions as shown in Fig. 2, which is consistent with the previous study^34^. The solubilization of Cd could be finished in 6 days with the removal rate of 64.36%. Cd was mainly present in mobile forms (91.07%) as shown in Fig. 3, which agreed with the findings of Zeng et al.^35^ Thus, the acid dissolution was the main removal mechanism of Cd^34^. Due to the low pH of the bioleached sludge, the content of Cd in mobile forms decreased by 62.77% after bioleaching. Furthermore, Cd in immobile forms (oxidizable and residual forms) also reduced significantly.

The previous study found that Cr was relatively stable with the dissolved pH threshold of 2.3–3.0^28^. Although the percentage of Cr present in mobile forms was over 40%, the removal rate of Cr (6.32%) was the lowest among all the heavy metals investigated as shown in Fig. 2, because the lowest pH of the bioleached sludge was about 2.9, which was close to the dissolution threshold limit of Cr.

As shown in Fig. 2, Mn and Ni were solubilized quickly in the first 4 days. The solubilization percentage of Mn and Ni were 56.14% and 49.83% after bioleaching, respectively. Mn and Ni mainly existed in the mobile forms (Mn 82.05%, Ni 76.08%). In the early stage of bioleaching, the removal rates of Mn and Ni were closely related to the variation of pH and displayed obvious acid dissolution mechanism. After bioleaching, the concentrations of Mn in exchangeable/acid soluble, reducible and oxidizable forms were reduced by 34.65%, 78.82% and 90.84%, respectively. As for Ni, the removal rates in such forms were 34.66%, 74.58% and 64.99%, respectively. Thus, the higher extraction efficiency of Mn and Ni arose from mixed bioleaching mechanisms, which contain acid dissolution, oxidation and reduction by Fe^2+^/Fe^3+^.

Relatively low removal efficiency of As (20.78%) was observed in this study. One reason, as shown in Fig. 3, was that As was mainly distributed in residual form with high stability. The other reason was that the dissolved As^3+^ could be oxidized to As^5+^ (AsO_4_^3-^) by Fe^3+^ generated from the metabolism of iron-oxidizing bacteria, and then insoluble FeAsO_4_ could be produced through the reaction of AsO_4_^3-^ and Fe^3+^, which resulted in the reprecipitation of As^34^.

Pb in exchangeable/acid soluble form was not detected in the raw sludge, and mainly existed in reducible (59.20%) and oxidizable (23.19%) forms. The removal rates of Pb in reducible and oxidizable forms were 33.51% and 58.17% after bioleaching, respectively. However, the insoluble compounds such as PbSO_4_ (K_sp_ = 1.62 × 10^–8^) could be generated during the bioleaching process^36^, which resulted in a significant increase in the concentration of Pb in residual form (from 10.89 to 25.00 mg/kg), and thus led to the low removal ratio of Pb (10.52%).

To summarize, the solubilization efficiencies of Zn, Cu, Cd, Mn and Ni, which mainly existed in mobile forms in the raw sludge, were relatively high due to the instability of these metals, while the removal rates of Cr, As and Pb, which mainly existed in immobile forms, were relatively low. However, the contents of most heavy metals in mobile forms decreased obviously after bioleaching and would lead to the corresponding reduction of the environmental risk of the sludge.

### Combined bioleaching/Fenton-like process

#### Effect of H_2_O_2_ dosage on the removal of heavy metals under various pH conditions

**Figure 4:**
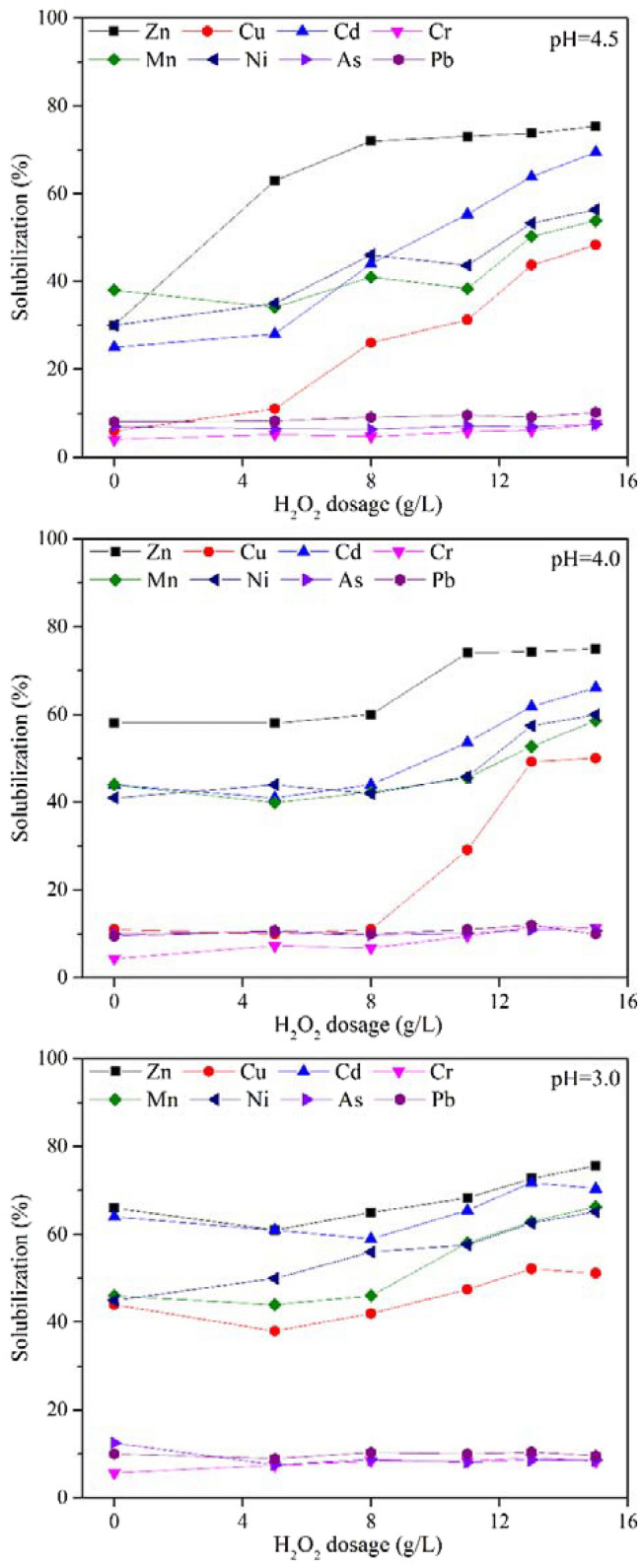
Effects of H_2_O_2_ dosage on the removal efficiency of heavy metals under various pH conditions.

Previous studies have shown that the production ability of hydroxyl radical during the Fenton-like reaction process could be enhanced under pH range of 2.5–4.5, and meanwhile, the amount of H_2_O_2_ directly influences the production of hydroxyl radical^10,18^. Therefore, as shown in Fig. 4, the effects of H_2_O_2_ dosage on the solubilization efficiencies of heavy metals were investigated at different stages of the bioleaching process, when the pH values of the bioleached sludge were 4.5 (about 3.5th day), 4.0 (4th day) and 3.0 (6th day).

With the increasing concentrations of H_2_O_2_ (0.0–8.0 g/L), the solubilization efficiency of Zn increased significantly at pH of 4.5 (Fig. 4) due to the oxidation of metal sulfide and organics by hydroxyl radical^10^. However, the solubilization percentages of Zn barely changed with further increase of H_2_O_2_ dosage (from 8.0 to 15.0 g/L). The solubilization percentage of Zn at the H_2_O_2_ dosage of 8.0 g/L (pH of 4.5) was significantly higher than when only using single bioleaching (75.31% vs. 67.64%). The enhancement of solubilization efficiency of Zn at a pH of 4.0 and 3.0 was not very noticeable (Fig. 4), because most of the Zn in immobile forms was dissolved out by bioleaching. The highest solubilization percentages of Zn were 74.96% at a pH of 4.0 and 75.53% at a pH of 3.0, which were 7.32% and 7.89% higher than that of the single bioleaching process.

Due to the lower dissolved pH threshold of Cu compared with Zn, the solubilization efficiency of Cu was significantly affected by the dosage of H_2_O_2_ at a pH of 4.5 and 4.0 as shown in Fig. 4, while when the reaction pH was 3.0, the subsequent Fenton treatment had a relatively small impact on the removal of Cu. The highest removal rate of Cu (52.17%) was obtained at pH of 3.0 and H_2_O_2_ dosage of 13.0 g/L, which was slightly higher than that of the single bioleaching (50.78%). The change in solubilization efficiency of Cd was similar to that of Cu. When the pH values were 4.5 and 4.0, the solubilization percentages of Cd with H_2_O_2_ dosage of 15.0 g/L were 4.59% and 1.23% higher than that of the single bioleaching process, respectively. Meanwhile, the highest solubilization percentage of Cd (71.91%) could be reached at a pH of 3.0 and H_2_O_2_ dosage of 13.0 g/L, which was higher than that of the single bioleaching process (64.86%).

The addition of H_2_O_2_ did not increase the removal rate of Cr significantly as shown in Fig. 4. At a reaction pH of 4.5, the solubilization percentage of Cr was 7.59% with H_2_O_2_ dosage of 15.0 g/L, which was a little higher than that of the single bioleaching process (6.32%), while the highest solubilization percentages of Cr could reach 11.63% and 9.18% at pH of 4.0 and 3.0, respectively, with H_2_O_2_ dosage of 15.0 g/L.

The solubilization process of Mn and Ni displayed similar trend as shown in Fig. 4. The solubilization percentage of Mn was not significantly improved when the H_2_O_2_ dosage was increased from 5.0 to 11.0 g/L at pH of 4.5 and 4.0, but a much faster increase of the removal rate was observed with the H_2_O_2_ dosage over 13.0 g/L. It could be due to the enhanced oxidizing ability of Fenton-like reaction with abundant H_2_O_2_. However, the solubilization efficiency of Mn under a pH of 3.0 began to increase with H_2_O_2_ concentration of 11.0 g/L, which could be attributed to the high efficiency of Fenton action under lower pH^15^. The highest removal percentage of Mn was 66.29% at pH of 3.0 and H_2_O_2_ dosage of 15.0 g/L, while the removal percentage of Mn in the single bioleaching process was 56.14%. The removal behavior of Ni at various pH was consistent with Mn. The highest removal rate of Ni (65.81%) was found at a pH of 3.0 with H_2_O_2_ dosage of 15.0 g/L, which was significantly improved, compared with the single bioleaching process (49.83%).

On the contrary, the removal efficiency of As and Pb in the combined process was not promoted compared with the single bioleaching process. Due to the strong oxidizing capacity of Fenton-like process, the yield of SO_4_^2−^ and insoluble FeAsO_4_ could be improved. Correspondingly, Pb^2+^ could be transformed into residual form, such as insoluble PbSO_4_^10^. Therefore, the removal efficiencies of As and Pb decreased in the combined process. The highest removal rates of As and Pb after Fenton-like treatment were 12.46% and 10.20%, respectively.

In the combined process, higher solubilization efficiencies of most heavy metals (Zn, Cu, Cd, Mn, Ni, Cr) could be achieved in 6 days. The removal efficiency of heavy metals (except Cr, As and Pb) of combined process (pH of 3.0, H_2_O_2_ dosage of 15 g/L) is higher than that of the single bioleaching process. The removal rate of Zn, Cu, Cd, Mn and Ni increased by 7.89%, 0.38%, 5.56%, 10.15% and 15.35%, respectively. Meanwhile, the total concentrations of heavy metals measured in this study after treatment could meet the control standards of pollutants in sludge for agricultural use of China (National Standard GB 4284-2018). The removal of As and Pb was not improved by the combined process, other methods such as chemical leaching, electrokinetic remediation and phytoremediation could be considered as alternatives. However, their transformation into insoluble forms may also reduce the bioavailability of heavy metals and increase the environmental safety of the treated sludge. For that reason, the chemical speciation distributions of heavy metals in the combined process were further analyzed in detail.

#### Chemical fraction distributions of heavy metals in the combined process

**Figure 5:**
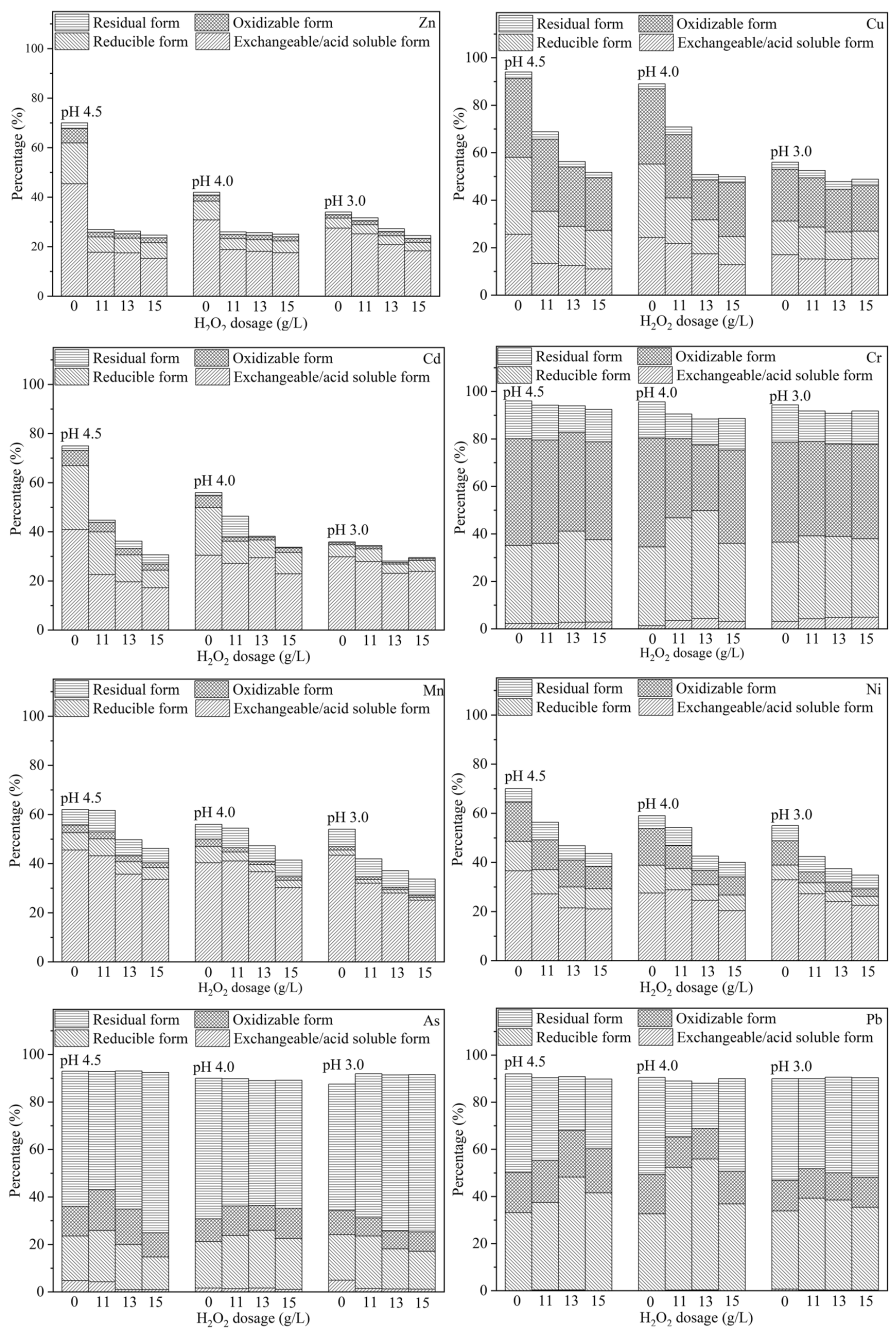
Change of chemical speciation distributions of heavy metals under different H_2_O_2_ dosage at a pH of 4.5, 4.0 and 3.0, total concentration of heavy metal in the raw sludge was set as 100%.

It can be seen in Fig. 4 that the solubilization efficiency of most heavy metals did not change significantly with H_2_O_2_ dosage below 8.0 g/L. Therefore, the chemical speciation changes of heavy metals after Fenton treatment under H_2_O_2_ dosage of 11.0, 13.0 and 15.0 g/L, as shown in Fig. 5, were discussed.

Under various pH conditions, the contents of Zn in all of the four forms showed a downward trend along with the increasing H_2_O_2_ dosage (Fig. 5). After bioleaching, Zn mainly existed in exchangeable/acid soluble form under the final pH of 4.5 (64.89%), pH of 4.0 (73.33%) and pH of 3.0 (80.82%). The removal of Zn in exchangeable/acid soluble form showed good correlation to the dosage of H_2_O_2_, which might be attributed to the destruction of EPS, and the released heavy metals were transferred to the liquid phase. Meanwhile, the improvement of sludge dewaterability could also promote the removal of heavy metals. After Fenton-like reaction at a pH of 4.5, the percentages of Zn in exchangeable/acid soluble forms were reduced by 30.35%, 31.41% and 40.09% at H_2_O_2_ dosage of 11.0, 13.0 and 15.0 g/L, respectively, compared with the percentage of Zn in the sludge at the end of the single bioleaching process. However, the percentage of Zn in other forms did not change significantly after Fenton-like treatment. Therefore, the further removal of Zn in exchangeable/acid soluble form and the dewaterability improvement of sludge may be the main reasons for the higher removal efficiency of Zn in the combined process.

Cu was still mainly associated with the oxidizable form after bioleaching ended at pH of 4.5, 4.0 and 3.0 (Fig. 5), which might be attributed to the preference of Cu for organic materials^22^. The addition of H_2_O_2_ at pH 4.5 significantly boosted the solubilization efficiency of Cu in exchangeable/acid soluble form. The percentages of Cu in exchangeable/acid soluble form in the sludge after Fenton treatment at pH 4.5 were 24.69% (11.0 g/L), 29.50% (13.0 g/L) and 38.15% (15.0 g/L), which were lower than that at the end of the single bioleaching process. Meanwhile, the content of Cu in reducible form was reduced by nearly 50% with H_2_O_2_ dosage of 13.0 and 15.0 g/L, compared with its content after bioleaching ended at pH 4.5. However, the highest removal rate of Cu in oxidizable form was only 33.20% with H_2_O_2_ dosage of 15.0 g/L. The removal efficiency of Cu in exchangeable/acid soluble and reducible forms increased with the increasing H_2_O_2_ dosage at pH 4.0 and 3.0, similar to the observation at pH 4.5. Under a reaction pH of 4.0, 47.2% of Cu in oxidizable form was removed after Fenton treatment with H_2_O_2_ dosage of 13.0 g/L, while only 28.6% was removed at H_2_O_2_ dosage of 15.0 g/L. In addition, the removal rates of Cu in oxidizable form were only 4.9–17.7% at various H_2_O_2_ dosage at a Fenton reaction pH of 3.0. The removal efficiency of Cu was reduced in despite of the increasing oxidation capacity of Fenton-like reaction. The macro-molecular organic matters could be degraded into small organic molecules during Fenton treatment process, releasing partial Cu. However, the generated small molecule organic matters had more undissociated carboxyl that would combine with released Cu^31^, which formed Cu in oxidizable form. Thus, it could explain the low removal efficiency of Cu in oxidizable form under stronger oxidizing condition. However, the highest removal rate of Cu (52.17%) was observed at pH 3.0 and H_2_O_2_ dosage of 15.0 g/L, due to the high reduction ratio of Cu in mobile forms at that condition.

Cd mainly existed in mobile forms in the sludge after bioleaching and Fenton treatment, as shown in Fig. 5. The contents of Cd in mobile and oxidizable forms decreased with the increasing H_2_O_2_ dosage at pH 4.5. The content of Cd in exchangeable/acid soluble form after Fenton treatment at pH 4.5 and H_2_O_2_ dosage of 15.0 g/L was 29.10% lower than that at the end of the single bioleaching process. Meanwhile, the content of Cd in mobile form was decreased by 27.54% (11.0 g/L), 26.56% (13.0 g/L) and 36.72% (15.0 g/L) after Fenton treatment at pH 4.0. The removal of Cd in exchangeable/acid soluble form after Fenton treatment could be largely due to the improvement of sludge dewaterability. However, the reduction of Cd was not obvious after Fenton treatment at pH 3.0, because the solubilization threshold of most of Cd in various forms were reached after the bioleaching process ended at pH 3.0.

The removal efficiency of Cr was not improved obviously by Fenton treatment in this study, as shown in Fig. 5. It was also reported that Cr was difficult to be removed by bioleaching or combined process due to its relatively high stability^10^. However, the content of Cr in oxidizable form after Fenton treatment at pH 4.5 was 4.76% (11.0 g/L), 9.20% (13.0 g/L) and 9.84% (15.0 g/L) lower than that at the end of the single bioleaching process, due to the strong oxidizing capacity of hydroxyl radical. And the lowest content of Cr in oxidizable form was observed after Fenton treatment at pH 4.0 and H_2_O_2_ dosages of 13.0 g/L, which was 39.4% lower than that in the bioleached sludge. Meanwhile, the highest Cr removal rate was also obtained at this condition after Fenton-like treatment. Thus, the improvement of Cr removal in combined process was mainly due to the release of Cr in oxidizable form. Furthermore, the released metals could be absorbed on the surface of oxides^31^, thus inevitably caused the increase of Cr in reducible form as shown in Fig. 5. The chemical speciation change of Cr after Fenton treatment at pH 3.0 was similar to that at pH 4.0.

The removal efficiency and chemical speciation distribution of Mn varied obviously after Fenton treatment with different dosages of H_2_O_2_. The removal rate of Mn was improved with the increasing dosage of H_2_O_2_ at various pH values. Because most of the Mn in reducible form (over 80%) was removed by bioleaching process, the reduction of Mn in exchangeable/acid soluble form should account for the removal of a substantial part of Mn after Fenton treatment. The highest removal rate of Mn in exchangeable/acid soluble form under different pH conditions was 26.27% (pH 4.5), 25.06% (pH 4.0) and 42.18% (pH 3.0), all with H_2_O_2_ dosage of 15.0 g/L. Although nearly 30% of Mn in reducible and oxidizable forms was also removed after Fenton treatment with H_2_O_2_ dosage of 15.0 g/L at various pH values, it contributed little to the removal of Mn considering the low concentration of Mn in reducible and oxidizable forms in the raw sludge. Furthermore, the changes of Mn in residual form were not obvious under different pH.

The chemical speciation change of Ni was similar to that of Mn after Fenton treatment. The contents of Ni in mobile and oxidizable forms decreased along with the increasing dosage of H_2_O_2,_ as shown in Fig. 5. Meanwhile, the reduction of Ni in exchangeable/acid soluble form after the addition of H_2_O_2_ was the prime reason for the higher removal efficiency of Ni after the combined process than that after the single bioleaching process. The highest removal rate of Ni in exchangeable/acid soluble form was found with H_2_O_2_ dosage of 15.0 g/L at pH 4.0, which was 34.47% lower than that in the sludge after the signal bioleaching process. However, the highest removal efficiency of Ni (65.19%) was reached when the reaction pH was 3.0 with H_2_O_2_ dosages of 15.0 g/L due to the simultaneous reduction of Ni in reducible and oxidizable forms. The contents of Ni in reducible and oxidizable forms were reduced by 50.30% and 52.83% under this reaction condition, respectively, compared with that at the end of the single bioleaching process.

As and Pb were mainly present in residual form before Fenton treatment as shown in Fig. 5. The content of As in exchangeable/acid soluble form decreased significantly due to the degradation of EPS at various pH values with the addition of H_2_O_2_. However, the content of As in residual form gradually rose with the increasing dosage of H_2_O_2_, probably because As^3+^ could be oxidized to As^5+^ by hydroxyl radical and/or Fe^3+^ with the formation of insoluble FeAsO_4_^34^. The content of Pb in reducible form showed a trend of increase after Fenton treatment. SO_4_^2−^ was generated due to the oxidation of sulfur elements and/or sulfide in sludge by hydroxyl radicals with the production of insoluble PbSO_4_^10^, and thus the content of Pb in residual form also increased after further Fenton treatment. Although the Fenton treatment had a negative impact on the removal of As and Pb as shown in Fig. 5, because of the formation of insoluble compounds under strong oxidizing condition, the environmental risk of these two heavy metals decreased to some extent under an appropriate condition, due to the increased proportion of immobile fractions, especially residual form. compared with the bioleached sludge.

The content and proportion of most heavy metals (Zn, Cu, Cd, Mn, Ni, As) in mobile forms were lower in the treated sludge after the combined bioleaching and Fenton-like process, compared with the single bioleaching process, which was also the main reason for the high removal efficiency of these metals. Their bioavailability and toxicity were also reduced. However, Fenton treatment was found to have a negative impact on the removal of As, but the increased proportion of As in residual form also lowered its bioavailability and mobility in the environment. The increase in the content of Pb in both mobile forms (mainly in reducible form) and immobile forms (mainly in residual form) was observed under different conditions, so special attention should be paid to the chemical speciation distributions of Pb during sludge treatment process.

### The effect of H_2_O_2_ dosage on sludge dewaterability at different pH values

**Figure 6:**
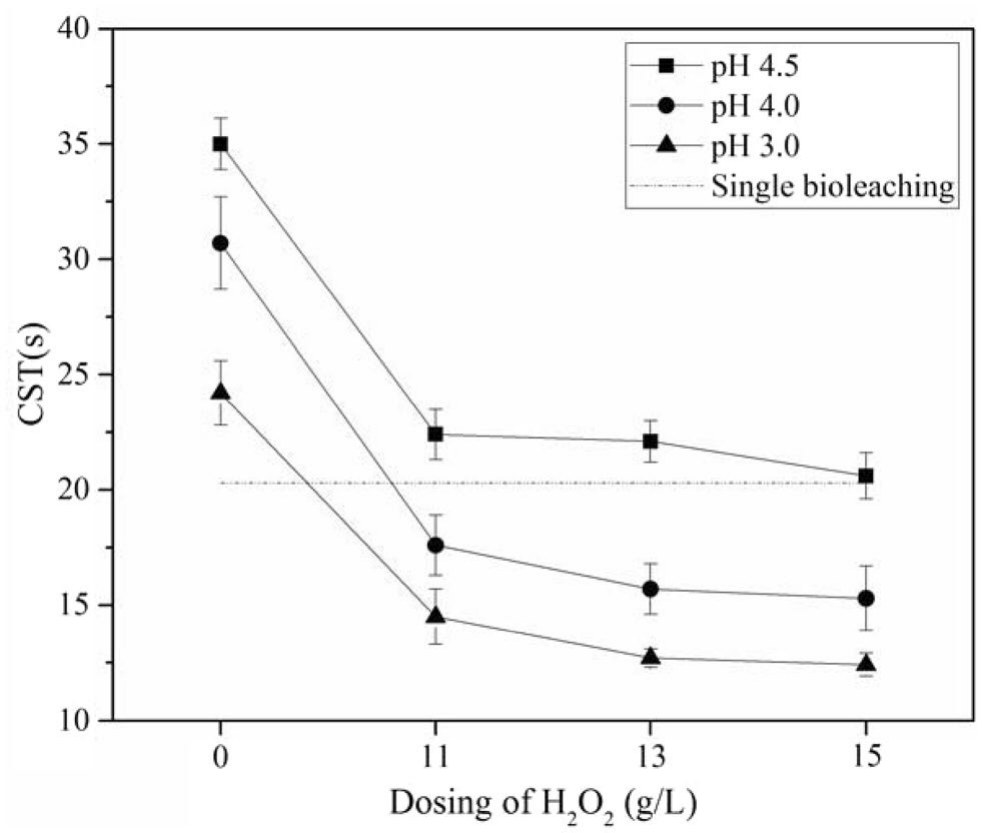
Changes of CST under different H_2_O_2_ dosage and pH.

The changes of CST of treated sludge under various conditions are presented in Fig. 6. The CST of the raw sludge (98.7 s) was dramatically reduced by bioleaching and Fenton oxidation treatments. After bioleaching ended on the 10th day (pH 2.89), the 6th day (pH 3.0), the 4th day (4.0) and the 3.5th day (pH 4.5), CST values of 20.3 s, 24.2 s, 30.7 s and 35.0 s were observed. The decreased pH after bioleaching process could destroy the EPS and neutralize the negative charge of the sludge flocs, resulting in the release of bound water^37^. Moreover, sludge dewatering could also be improved by the coagulation effect of Fe^2+^ ^10^. Furthermore, hydroxyl radicals were essential to improve sludge dewatering performance by destroying EPS and porous structure during the Fenton treatment process^35^. Therefore, the CST value of treated sludge was reduced to 20.6 s after Fenton treatment with H_2_O_2_ dosage of 15 g/L at pH 4.5, which was comparable to the CST value at the end of the single bioleaching process. The CST values were further reduced along with the decreasing reaction pH (4.0 and 3.0) and the increasing H_2_O_2_ dosage. The lowest CST value of 12.4 s was observed at Fenton reaction pH 3.0 and H_2_O_2_ dosage of 15.0 g/L, which meant a reduction from the initial CST of 87.44%. Therefore, the combined process could lead to an obvious improvement of the sludge dewaterability and significantly reduced the treatment period.

## Conclusion

Zn, Cu, Cd, Cr, Mn, Ni, As and Pb could be leached and removed to some extent after a 10-day bioleaching process. The contents of heavy metals in oxidizable form were all decreased. And the proportions of most heavy metals (except As) in mobile forms were also reduced after bioleaching. The pH value of the bioleached sludge could satisfy the requirement of Fenton-like process. The removal efficiencies of most heavy metals investigated (except As and Pb) in the combined process were improved under appropriate pH and H_2_O_2_ dosage, compared with that of the single bioleaching, due to the further removal of these heavy metals in mobile forms and oxidizable form after Fenton-like treatment. The combined bioleaching Fenton-like process could also significantly reduce the treatment time. However, the transformation of As and Pb into residual forms during Fenton treatment process had negative effects on their removal after the combined process. The reaction pH of Fenton and H_2_O_2_ dosage had obvious influence on the removal and chemical form transformation of the heavy metals. Meanwhile, the combined process also led to a significant improvement in the dewaterability of the treated sludge.
